# Long-term monitoring of Ca^2+^ dynamics in *C. elegans* pharynx: an *in vivo* energy balance sensor

**DOI:** 10.18632/oncotarget.12177

**Published:** 2016-09-21

**Authors:** Pilar Alvarez-Illera, Adolfo Sanchez-Blanco, Silvia Lopez-Burillo, Rosalba I. Fonteriz, Javier Alvarez, Mayte Montero

**Affiliations:** ^1^ Department of Biochemistry and Molecular Biology and Physiology, Institute of Biology and Molecular Genetics, Faculty of Medicine, University of Valladolid and CSIC, Ramón y Cajal, Valladolid, Spain; ^2^ Department of Biology, University of Hartford, West Hartford, Connecticut, USA

**Keywords:** calcium, pharynx, C. elegans, aging, energy balance, Gerotarget

## Abstract

Ca^2+^ is a key signal transducer for muscle contraction. Continuous *in vivo* monitoring of intracellular Ca^2+^-dynamics in *C. elegans* pharynx muscle revealed surprisingly complex Ca^2+^ patterns. Despite the age-dependent decline of pharynx pumping, we observed unaltered fast Ca^2+^ oscillations both in young and old worms. In addition, sporadic prolonged Ca^2+^ increases lasting many seconds or minutes were often observed in between periods of fast Ca^2+^ oscillations. We attribute them to the inhibition of ATP-dependent Ca^2+^-pumps upon energy depletion. Accordingly, food deprivation largely augmented the frequency of prolonged [Ca^2+^] increases. However, paradoxically, prolonged [Ca^2+^] increases were more frequently observed in young worms than in older ones, and less frequently observed in energy-deficient mitochondrial respiratory chain *nuo-6* mutants than in wild-type controls. We hypothesize that young animals are more susceptible to energy depletion due to their faster energy consumption rate, while *nuo-6* mutants may keep better the energy balance by slowing energy consumption. Our data therefore suggest that the metabolic state of the pharynx during feeding stimulation depends mainly on the delicate balance between the instant rates of energy production and consumption. Thus, *in vivo* monitoring of muscle Ca^2+^ dynamics can be used as a novel tool to study cellular energy availability.

## INTRODUCTION

The nematode *C. elegans* is one of the leading model organisms for aging research. One of the main advantages of this animal is the availability of well-described mutant strains with shortened or extended life spans, which provides a unique tool to dissect the molecular pathways related to aging [1]. Another major advantage is the possibility to visualize physiological alterations over the course of aging, as the normal *C. elegans* life span is only around two weeks. One of the physiological parameters that best correlates with aging is muscle activity. For example, *C. elegans* pharynx muscle activity has been shown to progressively decline with worm age [2-5].

The *C. elegans* pharynx is a neuromuscular organ that generates rhythmic contractions to grind the bacterial food and propel it into the digestive system. The worm pharynx has been in fact compared to the human heart, because it has similar electrical properties and its development is controlled by similar or homolog genes [6]. The rate of pharyngeal pumping reaches a maximum of nearly 300 pumps/min for 2 day-old adults, decreasing progressively with age to about 100 pumps/min by day 8, 10 pumps/min by day 12, and essentially no activity after day 14 [7]. Moreover, this pharyngeal pumping decline rate correlates with the longevity of worms. For example, *C. elegans* mutants with extended longevity show a significant delay in the rate of pharyngeal pumping decline. Likewise, *C. elegans* mutants with shortened longevity often display an accelerated decline of pharyngeal pumping [7]. Thus, pharyngeal muscle activity strongly correlates with aging as decline of pharyngeal pumping directly correlates with a decline in survival probability [7].

*C. elegans* pharynx contraction is due to repetitive Ca^2+^ transients induced by rhythmic action potentials, which qualitatively bear a resemblance to vertebrate cardiac action potentials [8]. Motoneuron stimulation triggers cell depolarization, which induces Ca^2+^ entry through two types of voltage-dependent Ca^2+^ channels in the plasma membrane of pharynx cells [9], and the subsequent cytosolic [Ca^2+^] increase is amplified by Ca^2+^ release from the sarcoplasmic reticulum through the ryanodine receptor [10]. Activation of a voltage-dependent potassium channels triggers a rapid repolarization that closes the plasma membrane Ca^2+^ channels, so that the Ca^2+^ pumps in the sarcoplasmic reticulum membrane and the plasma membrane pump back Ca^2+^ out of the cytosol, restoring the low resting [Ca^2+^]_c_ levels.

Ca^2+^ dynamics in *C. elegans* pharynx muscle have been previously measured using Ca^2+^-sensitive fluorescent proteins [11, 12]. However, the patterns of pharynx [Ca^2+^] oscillations in live worms during aging have never been studied. We were interested in obtaining detailed extended recordings of the pharynx muscle Ca^2+^ dynamics in worms at different times during the aging process. Our results show that, despite the obvious pharynx muscle decline observed during aging, Ca^2+^ oscillations do not undergo an age-dependent decline. In fact, similar fast Ca^2+^ oscillations could be obtained for both, young and physiologically active 2 day old worms as well as older and physiologically much less active 12 day old worms. Here we also report that the pattern of Ca^2+^ oscillations is strongly influenced by the availability of energy in the pharynx muscle cells, which in turn is determined by the balance between local energy production *versus* consumption. Our results suggest that imbalance due to excess energy consumption would lead to ATP depletion, which would result in the inhibition of Ca^2+^ pumps, leading to a sustained high [Ca^2+^]_c_. Thus, the new technique we show here to study long-lasting pharynx muscle Ca^2+^ dynamics may provide a novel methodology to *in vivo* monitor the energy status of the worm.

## RESULTS

### The patterns of Ca^2+^ dynamics in stimulated pharynx muscle include both fast Ca^2+^ spiking and “square-wave” [Ca^2+^]_c_ transients

The *C. elegans* pharynx is a neuromuscular organ that undergoes rhythmic contractions induced by oscillatory changes in cytosolic Ca^2+^ concentration ([Ca^2+^]_c_) [11, 12]. These muscle contractions are essential for worm survival, as they help to pump and grind food into the digestive system. Oscillatory [Ca^2+^]_c_ dynamics in the pharynx during pumping have been previously monitored with fluorescent Ca^2+^-sensitive protein probes targeted to the pharynx [11, 12]. However, only short (1-2 min) periods have been recorded, and correlations with worm age or with worm physiological conditions have not been attempted. We performed long (up to 30 min) continuous *in vivo* measurements of the *C. elegans* pharynx [Ca^2+^]_c_ dynamics, and studied the changes of the [Ca^2+^]_c_ patterns at different time points during worm aging.

**Figure 1:**
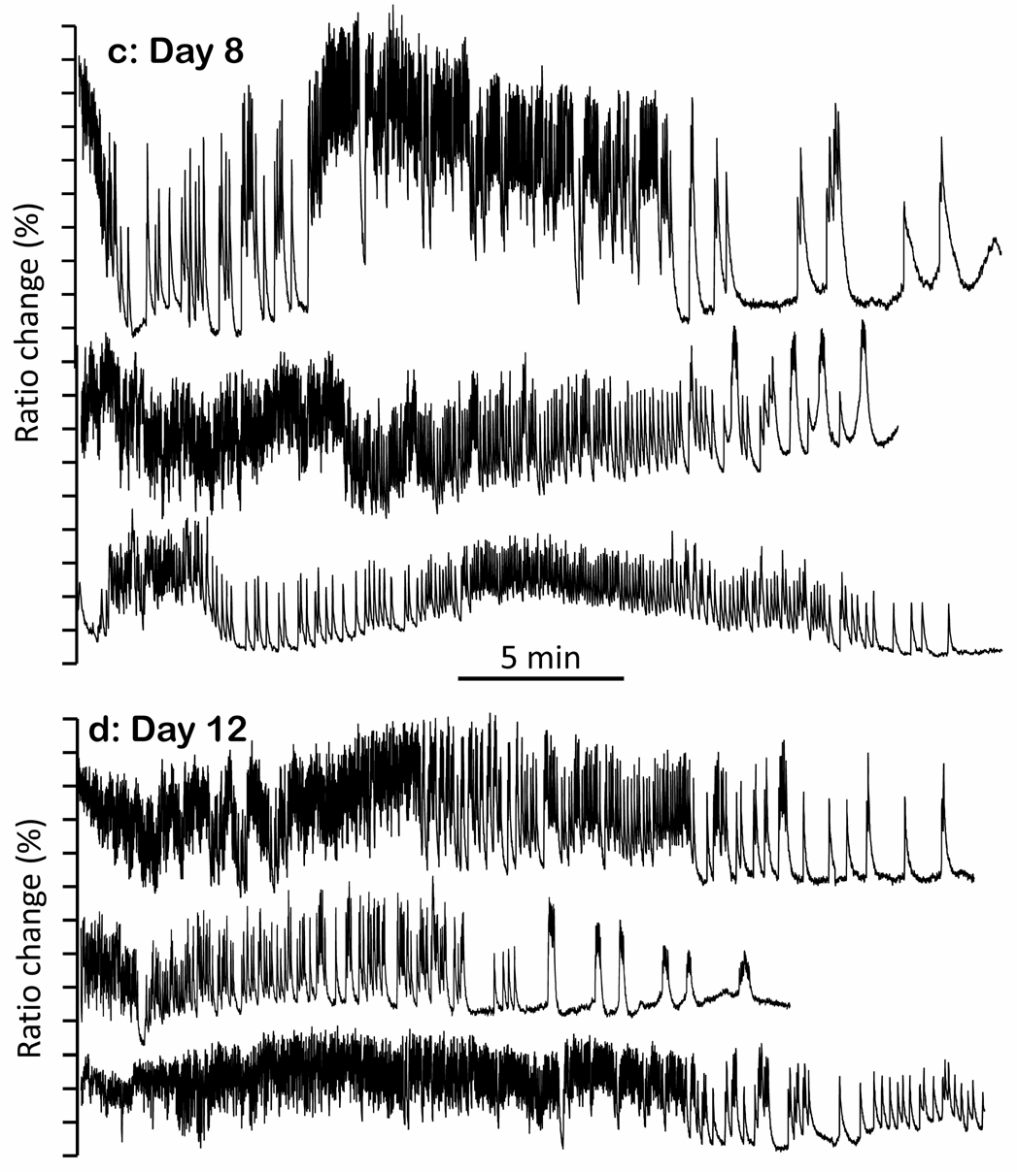
Representative traces of [Ca^2+^]_c_ dynamics in pharynx cytosol of *C. elegans* worms of 2 (panel a.), 5 (panel b.), 8 (panel c.) and 12 (panel d.) days old Each panel shows traces corresponding to 20-30 min activity of three representative worms of the corresponding age. In each worm, the region of the pharynx was graphically selected in the 535nm/480nm emission ratio image and the traces show the changes in the mean intensity of that region.

Remarkably, many of the worms we studied were able to keep a continuous [Ca^2+^]_c_ oscillatory activity for 30 minutes and even longer. Even though the frame recording frequency (1.5Hz) may have cut off some of the high frequency [Ca^2+^]_c_ changes, we were still able to detect a large variety of [Ca^2+^]_c_ spikes and patterns of [Ca^2+^]_c_ oscillation. We were interested in studying the [Ca^2+^]_c_ dynamics at different times during the life of the worms, thus we performed Ca^2+^-recording experiments with young (2 day old), mid-age (5 and 8 day old), and old worms (12 day old). Figure 1 shows representative individual recordings obtained at each of these ages. The data in our recordings revealed the complexity and individual variability of [Ca^2+^]_c_ signaling in live worms. Among the Ca^2+^ patterns that we observed, we distinguished two major types of [Ca^2+^]_c_ transients. The large majority of transients were comprised of relatively fast and repetitive [Ca^2+^]_c_ spikes, with rapid up and down phases. However, a few of the [Ca^2+^]_c_ transients were much more prolonged and had a “square-wave” shape, with a variable plateau phase of high [Ca^2+^]_c_ between the up and down phases (see Figure 1a).

The origin of these “square-wave” [Ca^2+^]_c_ transients is not clear. The function of the pharynx is to pump in and grind bacteria, thus a persistent contraction is not useful for that. In fact, repetitive contraction and relaxation is essential to make food advance to the intestine [13]. Moreover, a persistent increase in [Ca^2+^]_c_ is generally considered deleterious for the cells, leading to mitochondrial Ca^2+^ overload, activation of different hydrolytic enzymes, oxidative stress and cell death, as observed during ischemia/reperfusion in the myocardium [14]. In fact, physiological [Ca^2+^]_c_ signaling always tends to adopt the shape of single or repetitive [Ca^2+^]_c_ spikes. In these spikes, [Ca^2+^]_c_ increase from resting level is due to a transient activation of either Ca^2+^ entry from the extracellular medium, or Ca^2+^ release from intracellular stores. The subsequent rapid return of [Ca^2+^] to the resting level is assured mainly by ATP-dependent Ca^2+^ pumps, which are constantly operating provided there is enough ATP and [Ca^2+^]_c_ is high. Thus, Ca^2+^-dependent muscle contraction consumes large amounts of energy, partly due to contraction but also partly due to the continuous pumping back of Ca^2+^ into the sarcoplasmic reticulum and out of the cell. Therefore, transient or persistent shortage of cellular ATP appears to be the most probable explanation for these prolonged “square-wave” [Ca^2+^]_c_ peaks. ATP depletion would make contraction stop and would also lead to a prolonged high [Ca^2+^]_c_ due to the arrest of the Ca^2+^ pumps.

### Pharynx muscle cells of young worms are closer to energy depletion during stimulated contraction than those of older worms

Many of the recorded “square-wave” [Ca^2+^]_c_ increases were transient, whereby cells were able to take [Ca^2+^]_c_ back to resting levels and sometimes restart the fast spiking (Figure 1a). This suggests a reversible misbalance in the ATP production/consumption ratio. However, in some cases “square-wave” [Ca^2+^]_c_ transients lasted much longer, keeping [Ca^2+^]_c_ high for several minutes or even until the end of the experiment (Figure 1a and S2). In general, when the “square-wave” [Ca^2+^]_c_ transients were so long, little oscillatory [Ca^2+^]_c_ activity was observed afterwards, suggesting that irreversible depletion of energy could be the main reason for such prolonged increases in [Ca^2+^]_c_. It was therefore surprising to see that the “square-wave” [Ca^2+^]_c_ transients were much more frequent in young worms (day 2), which are known to be metabolically more active. In fact, it has been reported that the total *C. elegans* ATP reaches its maximum level when worms are 2-4 day old and then decreases progressively, so that only about 10% of the original ATP level is left by days 12-14 [15-18]. However, to our surprise the number of recorded “square-wave” [Ca^2+^]_c_ transients was highest at day 2 (Figure 1a), decreased considerably at days 5 and 8 (Figure 1b-1c), and almost disappeared by day 12 (Figure 1d).

The large number of [Ca^2+^]_c_ transients obtained from long and sustained (up to 30 minutes) [Ca^2+^]_c_ recordings acquired from more than 20 worms at four different ages, made it necessary to implement a custom-made computer analysis. When applied to the [Ca^2+^] records obtained from each worm, the program was able to quantify the height and the width of each [Ca^2+^] peak, as well as the spike frequency (see Methods). Following this approach we first analyzed the distribution of the [Ca^2+^]_c_ peak widths obtained from experiments performed with multiple individual worms of different ages, as we reasoned that the presence of the “square-wave” [Ca^2+^]_c_ increases would be best detected in that way.

**Figure 2:**
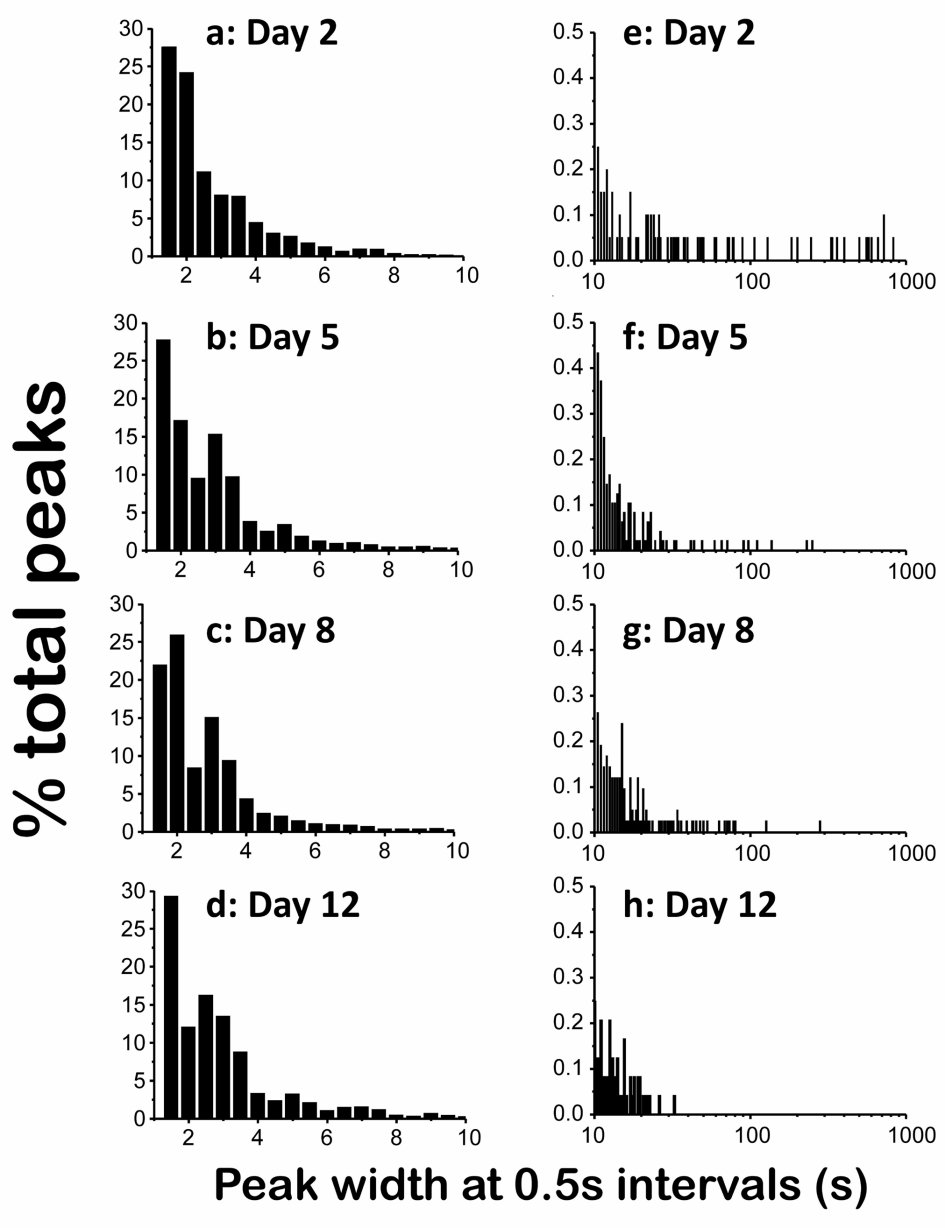
Distribution diagrams of the peak width in *C. elegans* worms of 2 (panels **a.** and **e.**), 5 (panels **b.** and **f.**), 8 (panels **c.** and **g.**) and 12 (panels **d.** and **h.**) days old Panels **a.**-**d.** show the percentage of peaks having widths smaller than 10s at 0,5s intervals. Panels **e.**-**h.** show the percentage of peaks having widths between 10 and 1000s, also with 0,5s intervals. Note the different scales.

Figure 2 shows the distribution of the widths of the peaks expressed as the percentage of peaks having a width in a certain interval, obtained by adding up the data of experiments performed with 24-28 worms of each age. As seen in the [Ca^2+^] records of Figure 1, most of the [Ca^2+^] spikes were involved in high-frequency [Ca^2+^] oscillations. Accordingly, about 50% of the [Ca^2+^] peaks at each age corresponded to fast spiking and had a narrow width, between 1s and 2s (Figure 2a-2d). For the rest of the peaks, the wider the peak the less frequent it was, and the frequency distribution corresponding to peaks having a width of up to 10s was very similar at all ages (Figure 2a-2d). However, the differences appeared when we studied the distribution at longer widths. Figure 2e-2h shows the tail of the distribution when we searched for peaks with widths beween 10s and 1000s, which correspond to those we previously called “square-wave” [Ca^2+^]_c_ transients. Each bar here corresponds to one “square-wave” [Ca^2+^]_c_ transient, and the position in the X axis indicates the width of the transient. Here it is quite clear that the number of long [Ca^2+^]_c_ transients was highest in young worms and decreased progressively with the age of the worm.

**Figure 3:**
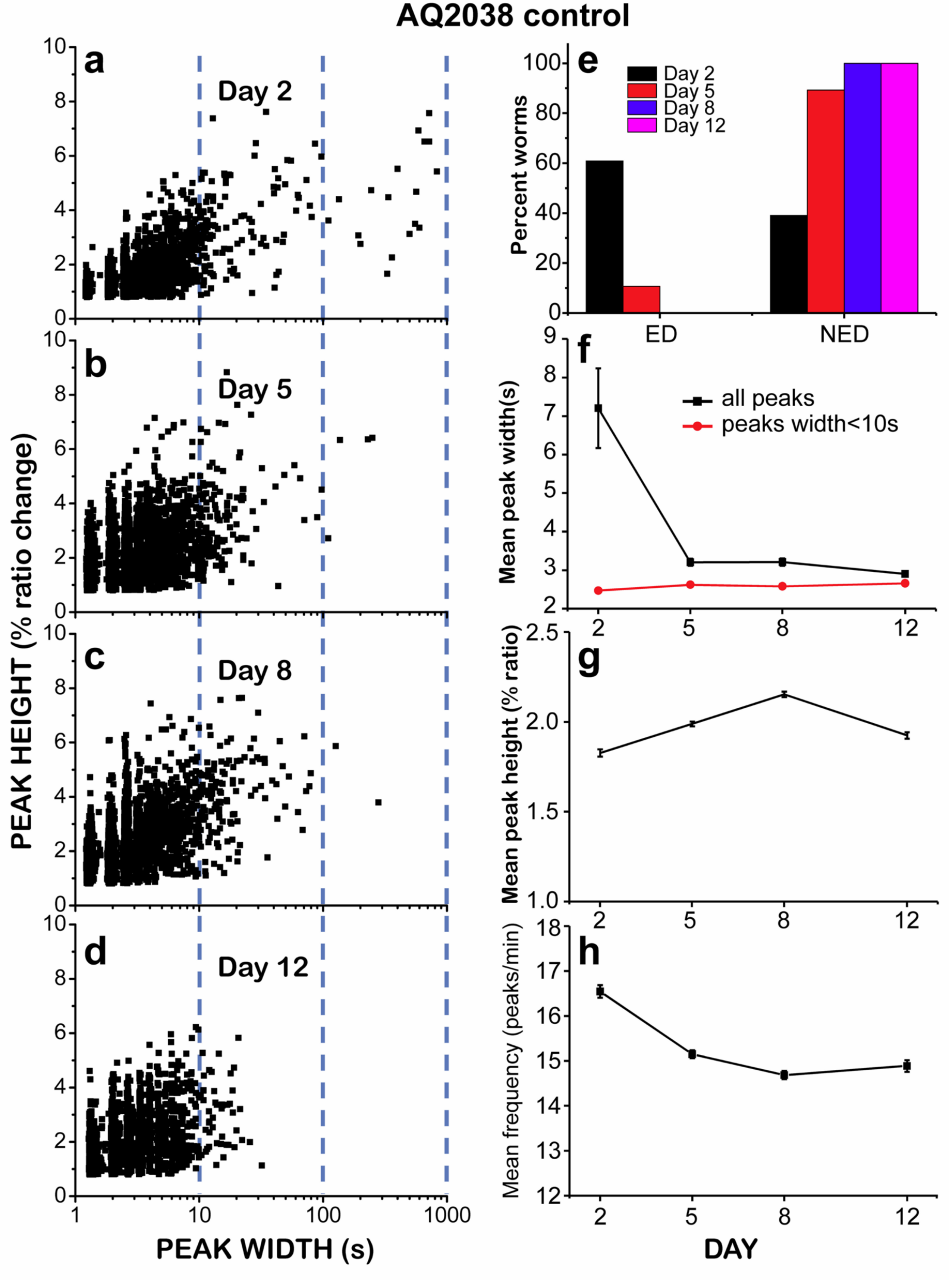
Analysis of [Ca^2+^]_c_ dynamics in *C. elegans* AQ2038 worms of 2, 5, 8 and 12 days old. Panels a.-d. show plots of peak height against peak width for all the peaks analyzed in worms of 2 (panel a., 2017 peaks from 24 worms studied), 5 (panel b., 4854 peaks from 28 worms studied), 8 (panel c., 4211 peaks from 28 worms studied) and 12 (panel d., 2412 peaks from 25 worms studied) days old Note the logarithmic scale in the peak widths. Panel **e.** shows the percent of worms classified as “Energy Depleted” (ED) or “Non Energy Depleted” (NED) at every day of worm life. Panel **f.** shows the variation in the mean peak width along the worm life, either including all the peaks or only those with width < 10s. Panels **g.** and **h.** show the variation in the mean peak height and the mean frequency, respectively, along the life of the worm. Data in panels f to h are mean±s.e.

The decline in the number of “square-wave” [Ca^2+^]_c_ transients during aging is also clearly seen in Figure 3a-3d, which shows plots of peak height against peak width, so that we can see every recorded [Ca^2+^]_c_ increase event as a single point in the plot. This representation shows a significant difference in the amount of long-lasting [Ca^2+^]_c_ peaks at different ages. Although most of the peaks have widths in the 1-10s interval at every age, long [Ca^2+^]_c_ transients having widths of more than 10s or even more than 100s were much more frequent in 2 day old worms (Figure 3a), and nearly disappeared in 12 day old worms (Figure 3d). This effect was also evident when we calculated the mean peak width, that is, the mean of all the widths of the [Ca^2+^] peaks recorded in worms of a given age (Figure 3f, black trace). Because of the large abundance of “square-wave” [Ca^2+^]_c_ transients in 2-day old worms, the mean peak width was much higher for them, about 7s, decreased to values around 3s for 5 and 8 day old worms, and decreased even further for 12 day old worms.

The mean peak width is largely influenced by the abundance of long “square-wave” [Ca^2+^]_c_ transients. To obtain a parameter more sensitive to the width of the fast spikes, we calculated the mean peak width after excluding all the [Ca^2+^]_c_ transients longer than 10s (mean peak width < 10) (Figure 3f, red trace). This parameter is a better representation of the fast oscillatory [Ca^2+^]_c_ kinetics and may reflect the ability of the cells to use energy to pump Ca^2+^ out of the cytosol, provided there is enough energy available. The faster the ATP is consumed for that, the faster the Ca^2+^ extrusion should happen and hence, the shorter the width of the spikes and the higher the frequency of [Ca^2+^] spiking. Figure 3f shows that the mean peak width (< 10) changed little during the whole life of the nematodes. Therefore, we find no change with age in the ability of the pharynx muscle to use energy to generate fast [Ca^2+^] spiking.

If the presence of “square-wave” [Ca^2+^]_c_ transients is due to lack of energy to reduce intracellular [Ca^2+^]_c_, we could classify the worms in two categories according to energy availability and depending on the way Ca^2+^ spiking activity ends. If Ca^2+^ activity ends in a “square-wave” [Ca^2+^] increase or a series of them, usually leaving [Ca^2+^] high at the end, we classified the worm as “Energy Depleted” (ED). Instead, if the spiking activity continued for more than 30 minutes or it ended before but in the form of narrow spikes, we assumed that the end of activity was due to other reasons and classified it as “Non Energy Depleted” (NED). Figure 3e shows that more than half of the worms of day 2 could be classified as ED, while only 10% of the worms of day 5, and essentially none of those of days 8 or 12 belonged to the ED type.

In addition of the width of the peaks, we also measured other parameters such as the height and the frequency of the Ca^2+^ spikes. These parameters changed less with aging. The mean height of the peaks changed less than 20% when worms got older (Figure 3g), and the frequency decreased only about 10% (Figure 3h). These two parameters are mainly related with the behavior of the fast repetitive spikes, which represent the large majority of the Ca^2+^ peaks. Therefore, the fast spikes, which correspond to the normal function of the pharynx muscle, triggering periodic pumping while there is enough energy available, appeared to be little modified during aging despite the progressive functional decline of pharynx contraction.

### Food deprivation of the worms accelerates energy depletion

**Figure 4:**
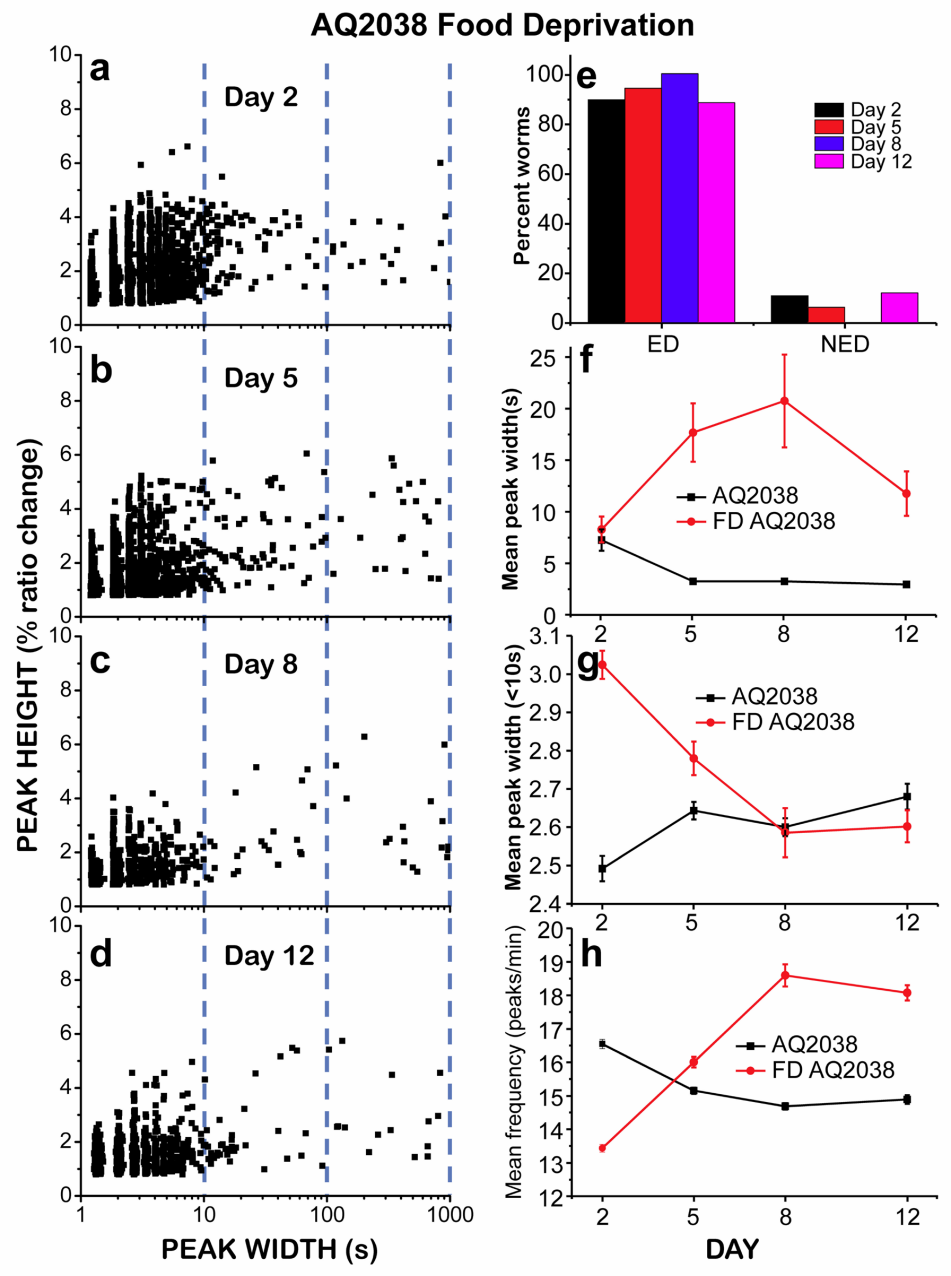
Analysis of [Ca^2+^]_c_ dynamics in *C. elegans* AQ2038 of 2, 5, 8 and 12 days old, after being under food deprivation (FD) since day 1 Panels a.-d. show plots of peak height against peak width for all the peaks analyzed in worms of 2 (panel a., 2239 peaks from 19 worms studied), 5 (panel b., 1527 peaks from 35 worms studied), 8 (panel c., 630 peaks from 14 worms studied) and 12 (panel d., 1295 peaks from 19 worms studied) days old. Panel e. shows the percent of worms classified as “Energy Depleted” (ED) or “Non Energy Depleted” (NED) at every day of worm life. Panel f. shows the variation in the mean peak width along the worm life, comparing AQ2038 controls with AQ2038 FD. Panel g. shows the variation in the mean peak width of peaks smaller than 10s along the worm life, comparing AQ2038 controls with AQ2038 FD. Panel h. shows the variation in the mean peak frequency along the life of the worm, comparing AQ2038 controls with AQ2038 FD. Data in panels f. to h. are mean±s.e.

If energy depletion was actually the reason for the development of “square-wave” [Ca^2+^]_c_ transients, then these prolonged [Ca^2+^]_c_ increases should appear with greater frequency in worms subjected to food deprivation. As previously reported, complete food deprivation extends *C. elegans* lifespan as long as partial dietary restriction [19] and in fact increases pharyngeal pumping at all ages [20]. This increase in energy expenditure together with the absence of food should accelerate the development of energy depletion. Our data supported this hypothesis, as we found that in food deprived worms there was a large increase in the number of “square-wave” [Ca^2+^]_c_ transients at every age (Figure 4a-4d). The height *versus* width plots showed the presence of a large number of “square-wave” [Ca^2+^]_c_ transients at all ages. The mean peak width was already higher than in the controls at day 2, and remained high during the whole life of the worms (Figure 4f). In fact, more than 90% of the starved worms at every age were classified as ED (Figure 4e), despite the fact that complete food deprivation actually extends the lifespan [19, 20]. Supplementary Figure S2 shows typical examples of recordings obtained in food deprived worms, showing that in most cases the recordings end with a persistent elevation of [Ca^2+^]_c_.

In food-deprived worms, the typical pattern of [Ca^2+^] spiking consisted in a 5-10 minute initial period of fast [Ca^2+^] spiking, followed in most cases by one or more “square-wave” [Ca^2+^]_c_ transients until the end of the recording (Figure S2). The effect of food deprivation on the initial period of fast Ca^2+^ spiking was investigated using the mean peak width (< 10s) and the mean frequency. Figure 4g-4h compare the changes in these parameters in control and food-deprived worms during aging. The mean peak width (< 10s) was about 20% larger in food-deprived worms than in controls at day 2, but the difference was smaller at day 5 and returned to the same values of the controls at days 8 and 12. Regarding the mean peak frequency, it usually mirrors the changes in the mean peak width (< 10s), because the larger is the peak width, the smaller should be the frequency of the fast Ca^2+^ spiking. In this case (Figure 4h), the frequency was smaller in food-deprived worms than in controls at day 2, but increased during aging to reach similar or higher values than in the controls. Therefore, fast [Ca^2+^] spiking keeps operating normally in food-deprived worms while there is enough energy available.

### *nuo-6* mitochondrial respiratory chain mutant worms are more resistant to energy depletion, despite their reduced energy availability

**Figure 5:**
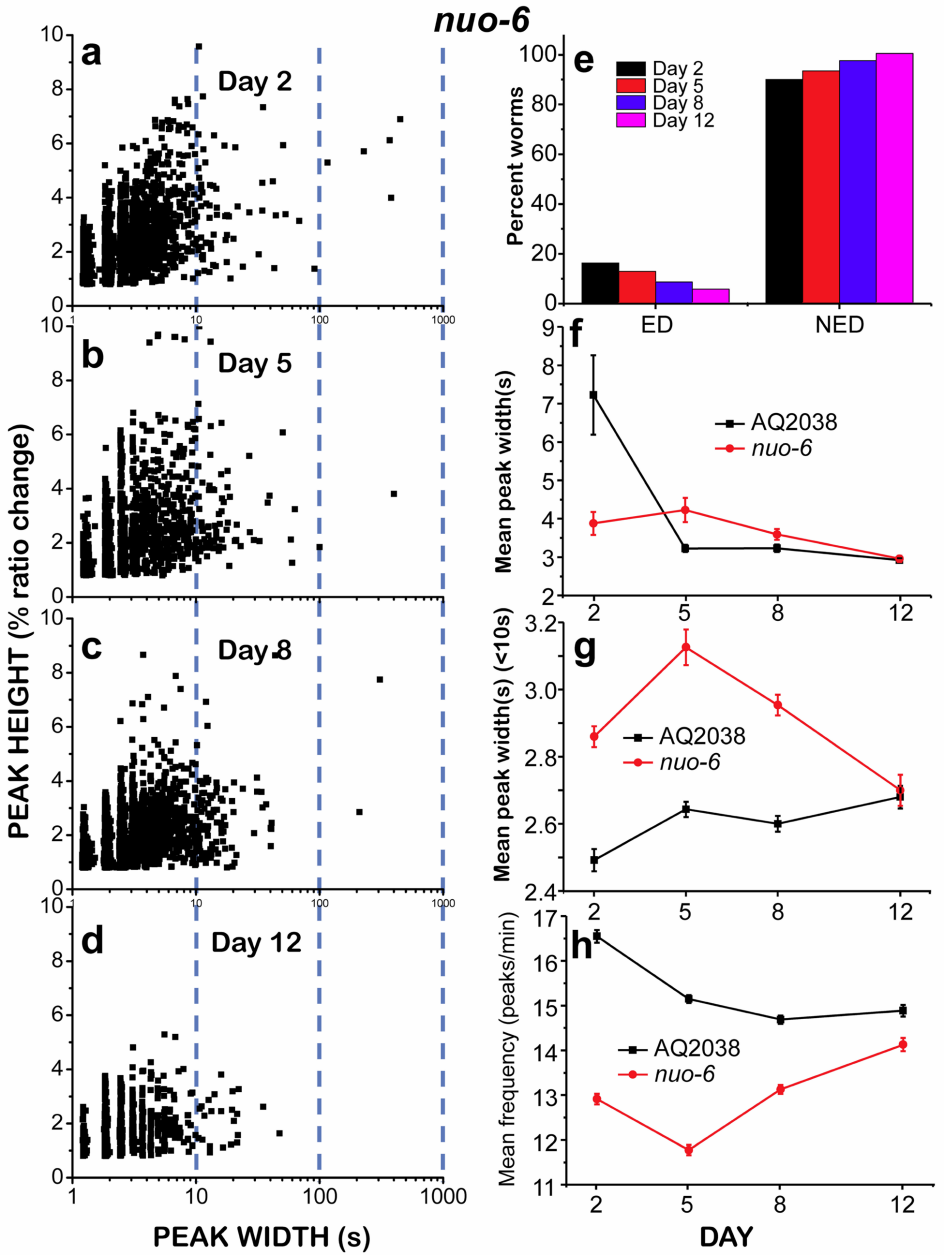
Analysis of [Ca^2+^]_c_ dynamics in *nuo-6* mutant worms of 2, 5, 8 and 12 days old Panels a.-d. show plots of peak height against peak width for all the peaks analyzed in worms of 2 (panel a., 2561 peaks from 43 worms studied), 5 (panel b., 1409 peaks from 19 worms studied), 8 (panel c., 2873 peaks from 41 worms studied) and 12 (panel d., 1241 peaks from 23 worms studied) days old. Other panels as in Figure 4. Data in panels f. to h. are mean±s.e.

We also studied pharynx muscle [Ca^2+^]_c_ dynamics in *nuo-6* mutant worms, which are defective in a subunit of complex I of the mitochondrial respiratory chain. *nuo-6* worms have reduced mitochondrial function and display lower oxygen consumption, slow growth, slow movement [21], decreased ATP levels [22] and a significant lifespan extension. The rationale to use this mutant was that if energy depletion was the reason for the “square-wave” [Ca^2+^]_c_ transients, this phenomenon should be enhanced in *nuo-6* worms due to their significantly reduced rate of ATP production. However, the results obtained were counterintuitive. Figure 5a-5d shows that there were few “square-wave” [Ca^2+^]_c_ transients in 2 day old *nuo-6* mutants. As observed in controls, their number decreased further when the worms aged. However, when we looked at the evolution of the total mean width of the [Ca^2+^]_c_ transients during aging (Figure 5f), we found that the dynamics was completely different from that observed in controls. The mean peak width in 2 day old worms was lower than in controls, but then remained nearly constant in 5-day old worms, and only decreased slightly in day 8 and day 12 old worms. Therefore, the proportion of “square-wave” [Ca^2+^]_c_ transients in young *nuo-6* mutant worms was much smaller than that of controls, and their contribution to the mean peak width was marginal. Figure S3 shows [Ca^2+^] recordings obtained in three 2-day old *nuo-6* worms, showing the presence of few “square-wave” [Ca^2+^]_c_ transients mixed with fast [Ca^2+^] spiking of various frequencies.

We then studied the kinetics of the fast [Ca^2+^] spiking in *nuo-6* mutant worms by measuring the mean peak width (< 10s) and the mean peak frequency (Figure 5g-5h) during aging. It was remarkable that the mean peak width (< 10s) in *nuo-6* mutant worms was much higher than in controls, particularly in young worms (Figure 5g). Accordingly, the mean peak frequency was smaller in *nuo-6* worms than in controls at all time points (Figure 5h). This suggests that the amount of ATP available in the *nuo-6* mutants is smaller at every age, thus the reduced ATP concentration would slow Ca^2+^ pumping out of the cytosol. However, in spite of the lower energy availability and the reduced energy production, we observed less frequent “square-wave” [Ca^2+^]_c_ transients. Similarly, when we classified the worms as ED or NED (Figure 5e), we found that the number of *nuo-6* mutant worms undergoing ED was below 20% at every age, in contrast with the results obtained for the controls (Figure 3e).

**Figure 6:**
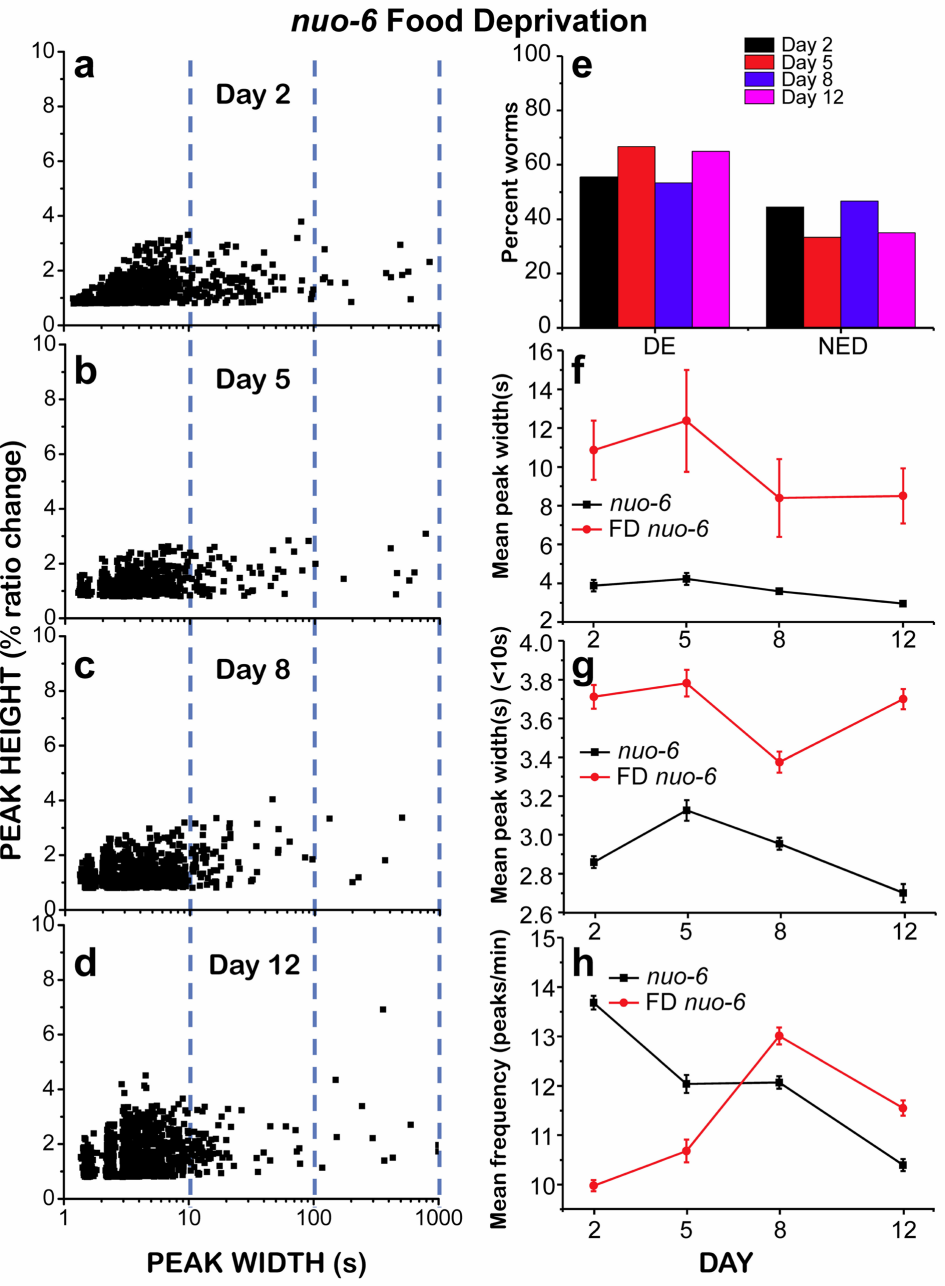
Analysis of [Ca^2+^]_c_ dynamics in *nuo-6* mutant worms of 2, 5, 8 and 12 days old, after being under food deprivation (FD) since day 1 Panels **a.**-**d.** show plots of peak height against peak width for all the peaks analyzed in worms of 2 (panel **a.**, 1013 peaks from 18 worms studied), 5 (panel **b.**, 681 peaks from 12 worms studied), 8 (panel **c.**, 1086 peaks from 15 worms studied) and 12 (panel **d.**, 1216 peaks from 20 worms studied) days old. Other panels as in Figure 4. Data in panels **f.** to **h.** are mean±s.e.

Given that *nuo-6* mutant worms were apparently less susceptible to undergo “square-wave” [Ca^2+^]_c_ transients, we decided to study if we could induce the development of “square-wave” [Ca^2+^]_c_ transients in these mutants by food deprivation, as we did for wild type worms. That was the case, although the number of “square-wave” [Ca^2+^]_c_ transients and energy depleted worms for *nuo-6* mutants was still much smaller than for food-deprived wild type worms (compare Figures 4 and 6). In food-deprived *nuo-6* worms, the total mean peak width and the number of ED worms were largely increased during the entire life of the worms (Figure 6e and 6f). However, nearly 50% of the worms were still able to maintain a persistent fast [Ca^2+^] spiking at every age. Moreover, the mean peak width (< 10) was significantly increased during the whole life of the worms with respect to fed *nuo-6* mutants (Figure 6g) and the frequency was smaller, particularly in the first days (Figure 6h). Therefore, food deprivation of *nuo-6* mutants further decreases the amount of energy available for Ca^2+^ pumping, but somehow they are less prone to reach full energy depletion than wild type controls.

**Figure 7:**
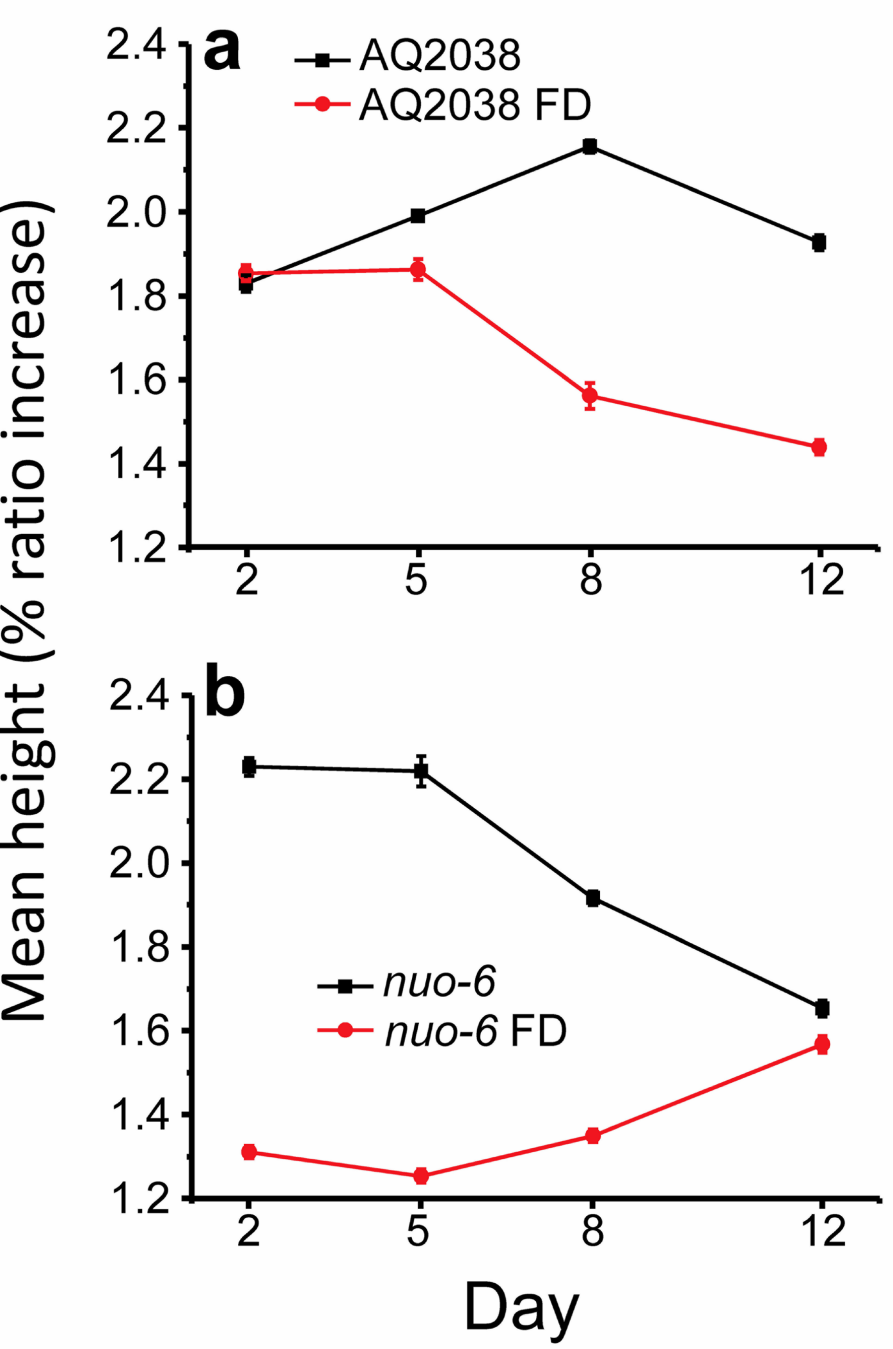
Variation of the mean height of the peaks with the worm age Panel **a.** compares AQ2038 controls with AQ2038 under food deprivation (FD) and panel **b.** compares *nuo-6* mutants with *nuo-6* under food deprivation (FD). Data are mean±s.e.

Finally, Figure 7 shows the changes in the height of the [Ca^2+^]_c_ peaks under the different conditions assayed in this work. The height of the peaks mainly depends of the magnitude of the stimulation, either Ca^2+^ entry from the extracellular medium or Ca^2+^ release from intracellular stores. As mentioned in the introduction, motoneuron stimulation of pharynx muscle triggers cell depolarization, which induces Ca^2+^ entry through two types of voltage-dependent Ca^2+^ channels, which is further amplified by Ca^2+^ release from the sarcoplasmic reticulum. As shown in Figure 3, there were not many changes in the mean height of the peaks during aging in wild type controls. However, increased energy depletion was generally associated with a decrease in the magnitude of the peaks. This occurs in food-deprived worms compared with the corresponding ad libitum fed controls, both in wild type (Figure 7a) and in *nuo-6* mutant worms (Figure 7b). This phenomenon may be explained by an impaired refilling of the sarcoplasmic reticulum with Ca^2+^ caused by the lack of energy, which would in turn lead to a partial Ca^2+^ depletion of this organelle. Sarcoplasmic reticulum Ca^2+^ depletion should lead to a progressive reduction in the amount of Ca^2+^ released in each oscillation and therefore a decrease in the height of the peaks. Obviously, this implies also a reduction in the amount of ATP consumption for Ca^2+^ pumping under these conditions.

## DISCUSSION

The *C. elegans* pharynx is a neuromuscular organ that undergoes rhythmic contractions in order to facilitate food ingestion. The maximum rate of pharyngeal pumping is achieved in two-day-old adults and reaches values of up to 300 pumps per minute. The pharyngeal pumping rate undergoes a progressive decline during aging and typically ceases when worms reach around 12 days of age [7, 23]. Moreover, this pharyngeal pumping decline rate correlates with worm longevity [7]. Because of the strong correlation between pharyngeal pumping rates and longevity, we decided to study Ca^2+^ dynamics in pharynx muscle as a way to investigate new functional parameters related to the aging process. Our results revealed several unexpected findings.

First, unlike the well documented pharyngeal pumping decline that worms experience over time, the fast Ca^2+^ spiking activity in the pharynx muscle did not undergo a progressive decline with age. Both the width and the height of the fast Ca^2+^ spikes remained virtually unchanged during aging, and just a 10% decrease in the mean frequency of the Ca^2+^ peaks was observed between young and old worms. Therefore, we find that aging induces a clear dissociation among muscle contraction and the Ca^2+^ signal that triggers contraction. This suggests that aging affects much more to the phenomenon of muscle contraction, the physical binding of myosin and actin to develop force, than to the development of the [Ca^2+^]_c_ signal required to induce contraction. In old animals, the [Ca^2+^]_c_ signal in the pharynx muscle cells remains essentially intact despite the fact that pharynx muscle is barely contracting. This is consistent with previous data indicating that sarcopenia is the major cause of aging-related functional decline in muscles, and that contraction-related injury may significantly promote the progression of sarcopenia in the pharynx [4]. Instead, the nervous system appears to be much better preserved with age [5]. Therefore, it appears that the machinery required to generate oscillatory [Ca^2+^]_c_ activity in the pharynx muscle is preserved in old adults, even after the contractile proteins stopped being functional.

A second unexpected finding involves the formation of “square-wave” [Ca^2+^]_c_ transients. Such prolonged elevations of Ca^2+^ are ineffective for food ingestion because they would just induce a persistent contraction of the pharynx. In addition, prolonged elevations of [Ca^2+^]_c_ are generally deleterious for the cells and imply a poor function of the mechanism responsible for Ca^2+^ pumping, either out of the cell or into the sarcoplasmic reticulum. Cells with normal function display global [Ca^2+^]_c_ elevations in the cytosol, which are always transient. The mechanisms that take Ca^2+^ out of the cytosol (pumps and exchangers) are always active, while the mechanisms that introduce Ca^2+^ in the cytosol (channels) are activated in a transient form. Therefore, “square-wave” [Ca^2+^]_c_ transients, whereby [Ca^2+^]_c_ is kept high for minutes at a time, can only be attributed to the blocking of the Ca^2+^ pumps responsible for clearing [Ca^2+^]_c_. We hypothesize that this phenomenon would be produced by the lack of ATP, which would prevent Ca^2+^ pumping activity.

To investigate whether ATP depletion was the cause of the “square-wave” [Ca^2+^]_c_ transients, we studied worms that were subjected to food deprivation. It has been reported that complete food deprivation extends *C. elegans* lifespan [19, 20] and leads to increased pumping rates and average locomotion compared to same age *ad libitum* fed worms [20]. It has also been shown that the metabolic rate is not reduced in food restricted worms [24], even though this condition should significantly limit the total amount of energy available. Therefore, the combination of increased pumping and locomotion activity as well as the sustained metabolic rate should lead to earlier energy depletion in pharynx muscle. Accordingly, the number of “square-wave” [Ca^2+^]_c_ transients was largely increased by this treatment not only in young adults but also during the entire life of the worms. Therefore, these data supports our hypothesis that the lack of energy may be a key determinant for the appearance of “square-wave” [Ca^2+^]_c_ transients.

Paradoxically, the highest number of “square-wave” [Ca^2+^]_c_ transients in wild type ad libitum fed worms was obtained when the nematodes were 2 days of age. This was a counterintuitive result as a priori we would not expect ATP depletion or cell deterioration for these young worms. Instead, the number of “square-wave” [Ca^2+^]_c_ transients was largely decreased by day 12. It has been shown that the ATP content in wild-type *C. elegans* worms reaches its maximum when worms are 2-4 days of age, and then decreases progressively until day 12-14 [15-18]. The total amount of ATP is thus much higher in younger nematodes than in older ones. Therefore, if energy depletion is the reason for the development of “square-wave” [Ca^2+^]_c_ transients, it seems contradictory that they appear only in young wild-type adults and then progressively disappear as they get older.

Our interpretation of this paradox is based in the concept of energy balance. In the case of young adults, energy depletion could come from a very high energy use rate, which would overwhelm the energy production rate. In all our Ca^2+^-recording experiments, worm feeding behavior was stimulated with serotonin [25, 26]. In the presence of serotonin, the Ca^2+^ oscillatory activity in the pharynx is rapidly induced [11, 12]. In young worms, the fast [Ca^2+^] oscillations lead to a fast pumping rate and repetitive contraction, which uses large amounts of ATP. For many instances, this leads to depletion of the cellular energy stores during the 30-min time of the experiment. As worms become older, the Ca^2+^ oscillatory activity remains constant, but the pharynx pumping rate decreases and so does the amount of energy dedicated to contraction. In fact, Ca^2+^ oscillatory activity remains nearly intact in 12-day old worms but these display minimal pharynx muscle contraction. We believe that the lack of contraction reduces substantially the amount of energy used by the pharynx muscle cells and this may allow the worm to maintain Ca^2+^ oscillations.

The data obtained in the *nuo-6* mutant worms were also paradoxical, but could again be explained on the basis of the energy balance hypothesis. These mutant worms have a lower ATP content than wild-type worms [22], but showed very few “square-wave” [Ca^2+^]_c_ transients at any age. The pumping rate of these mutant worms has been shown to be much smaller than that of wild-type worms [21], and they also show low oxygen consumption, slow growth, slow behavior, and increased lifespan [21, 22]. We propose that since *nuo-6* mutants display a low pumping rate and slow energy-consuming processes in general [21, 22], their smaller rate of energy use would allow them to keep a better balance between energy production and consumption in spite of their lower rate of energy production. This hypothesis would explain why *nuo-6* worms are less prone to reach energy depletion and thus show fewer “square-wave” [Ca^2+^]_c_ transients.

The balance between energy production and consumption becomes therefore essential to avoid energy depletion of pharynx muscle cells and the subsequent deleterious effects as consequence of the persistent increase in [Ca^2+^]_c_. This balance was again altered when *nuo-6* worms were food deprived. Under these conditions, the lack of energy production caused by the lack of food rapidly leads to ATP depletion and to a significant increase in the number of “square-wave” [Ca^2+^]_c_ transients at all ages. The lower ATP levels of *nuo-6* mutant worms may also be the reason for the increase in the width of the fast spikes (mean peak width < 10s) of the mutant worms. These fast spikes mainly depend on the rate of Ca^2+^ pumping out of the cytosol, either out of the cell or into the sarcoplasmic reticulum. Accordingly, the mean peak width (< 10s) was further increased in food-deprived *nuo-6* mutant worms.

In conclusion, the new technique we show here to study long-lasting pharynx muscle Ca^2+^ dynamics allows to monitor *in vivo* the energy status of the worms and provides new information on the metabolic state of the pharynx muscle cells. We show that the balance between energy production and consumption determines the ability of the worm to cope with stress situations such as those induced by persistent feeding stimulation with serotonin. In young worms, their fast rate of energy use for both, muscle contraction as well as Ca^2+^-pumping, overwhelms their energy balance and leads them to energy depletion. In older worms the balance between energy production and consumption is restored despite their slower energy production and lower total energy available. Thus, pharynx muscle cells in older worms do not undergo full energy depletion.

The same phenomenon occurs in the energy-deficient mitochondrial respiratory chain *nuo-6* mutant worms (even in young worms) due to the lower rate of energy use of these mutants. Uncoupling of energy production and consumption through decreased energy utilization, the so-called reduced “rate of living”, has been proposed before [15, 27] as a mechanism to explain the enhanced life span of several long-lived *C. elegans* mutants. Our data support the “rate of living” hypothesis and suggest that reduced energy consumption in *nuo-6* mutant worms may allow them to survive under stress conditions that would otherwise generate energy depletion and persistent intracellular Ca^2+^ increase, which would in turn lead to mitochondrial Ca^2+^ overload and irreversible cell damage.

## MATERIALS AND METHODS

### *C. elegans* strains and maintenance

Strains used were: AQ2038, integrated strain expressing cytosolic cameleon 2.1. (YC2.1) on pharynx due to the promoter sequence of the myo-2 gene (*pmyo-2::YC2.1*) [28-30], kindly provided by Drs. Robyn Branicky and W. Schafer, MRC Laboratory of Molecular Biology, Cambridge, UK. Mutant *nuo-6* strains expressing the Ca^2+^-sensitive protein were obtained by crossing AQ2038 worms with *nuo-6* (qm200) [21], obtained from the Caenorhabditis Genetics Center.

Worms were maintained and handled as previously described [31]. Hardened agar was seeded with *Escherichia coli* (OP50) and all the strains were maintained at 20°C. Calcium imaging experiments were also conducted at 20°C. Experiments were carried out on synchronized worms. Eggs were obtained as described previously [31] and were transferred to *E. coli* (OP50) seeded NGM plates. Young adults (day 1) were transferred to *E. coli* (OP50) seeded NGM plates with 15μM FUDR to avoid progeny.

### Calcium imaging

Pharynx Ca^2+^ measurements were carried out at days 2, 5, 8 and 12 of worm life. Worms were first starved for 4 - 6 hours before the experiments. Then, the nematodes were glued (using Dermabond Topical Skin Adhesive, Johnson & Johnson) on an agar pad (2% agar in M9 buffer) and feeding behavior was stimulated by adding 5mg/ml of serotonin [11, 12, 25, 26]. The agar pad containing the glued worms was mounted in a chamber in the stage of a Zeiss Axiovert 200 inverted microscope. Fluorescence was excited at 430 nm using a Cairn monochromator (10nm bandwidth, continuous excitation) and images of the emitted fluorescence obtained with a 40x LD-A-Plan objective were collected using a 450nm long pass dichroic mirror and a Cairn Optosplit II emission image splitter to obtain separate images at 480nm and 535nm emission. The splitter contained emission filters DC/ET480/40m and DC/ET535/30m, and the dichroic mirror FF509-FDi01-25×36 (all from Chroma Technology). Simultaneous 500ms images at the two emission wavelengths were recorded continuously (1.5Hz image rate) by a Hamamatsu ORCA-ER camera, and a ratio image was obtained by dividing that obtained at 535nm emission by that obtained at 480nm emission. Data obtained at both wavelengths were checked to assure that peaks in the ratio always corresponded to inverted changes in the individual fluorescences (Figure S1). Experiments were performed at 20°C and carried on during 30 minutes of continuous recording.

### Data analysis

Fluorescence records were analyzed using the Metafluor program (Universal Imaging). The traces shown were obtained as the ratio among the image obtained at 535nm emission and that obtained at 480nm emission. Ratio was only considered acceptable when mirror changes at both wavelengths were clearly present (Figure S1). Fluorescence intensities and ratio changes were then analyzed with a specific algorithm designed to calculate off-line the width at mid-height expressed in seconds, the height obtained as percent of ratio change and the frequency of all the Ca^2+^ peaks in each experiment. The frequency was measured at each peak as 9 divided by the distance among the peak 4 positions before and the peak 4 positions after. The mean frequency was calculated as the mean of all the individual frequencies higher than 5 peaks/min obtained in worms of a given age and condition.

## SUPPLEMENTARY MATERIALS FIGURES


