# Supplementary material for: Long-term monitoring of Ca2+ dynamics in C. elegans pharynx: an in vivo energy balance sensor
Source: Oncotarget. 2016 Sep 21;7(42):67732–47. doi: 10.18632/oncotarget.12177 (PMC5356515; doi:10.18632/oncotarget.12177)
Supplement: Supplementary file 1 [file oncotarget-07-67732-s001.pdf]

Long-term monitoring of Ca^{2+} dynamics in *C. elegans* pharynx: an *in vivo* energy balance sensor

Supplementary Material

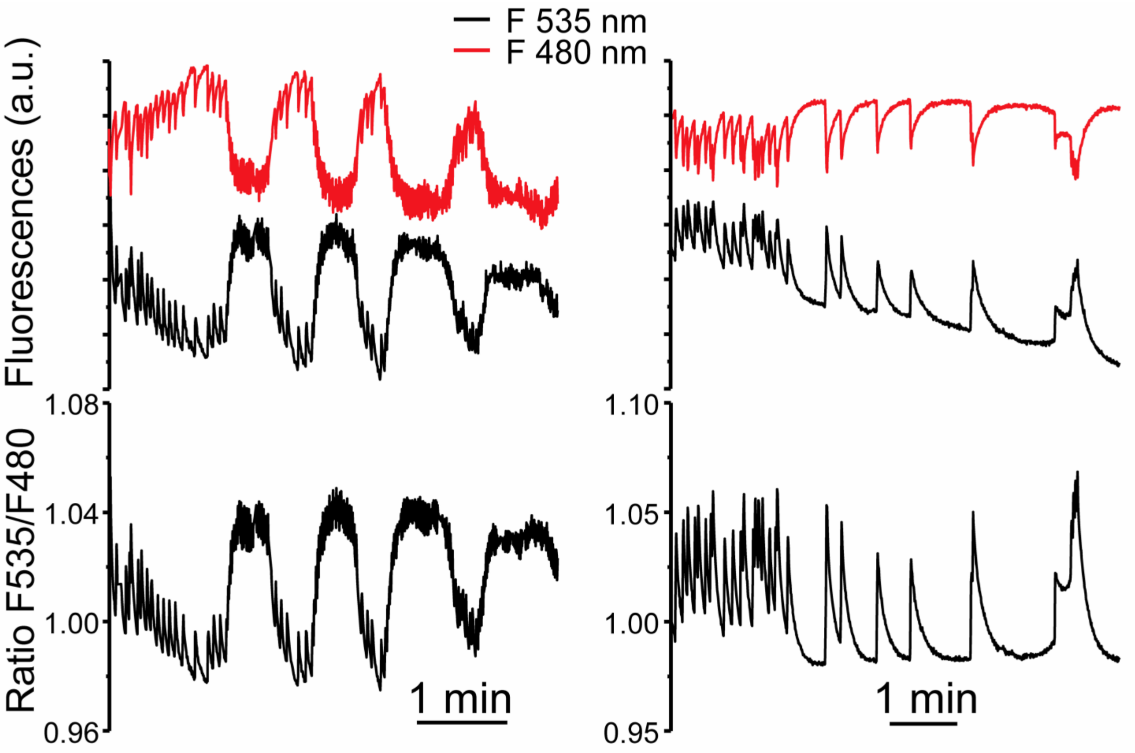

Figure S1. Simultaneous fluorescences obtained at 480nm and 535nm emission through the Cairn Optosplit II. The upper panels show the single fluorescences obtained at 480 and 535nm emission corresponding to two different experiments performed with AQ2038 worms of 8 days of age. The lower panels show the ratio F535/F480 in each case. Note that traces at each fluorescence mirror the changes observed in the other fluorescence.

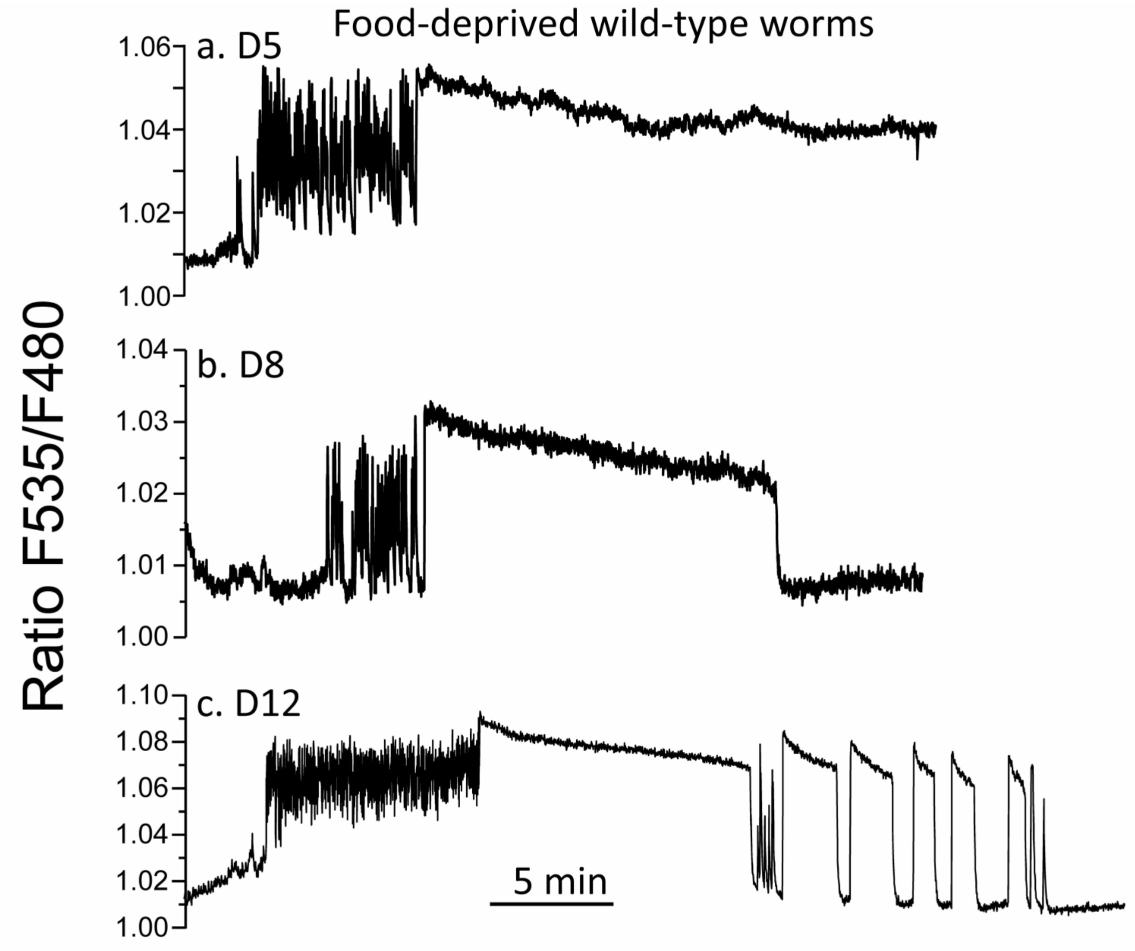

Figure S2. Representative examples of pharynx $[Ca^{2+}]_c$ recordings indicating energy depletion. They correspond to 5, 8 and 12 day-old AQ2038 worms under food deprivation.

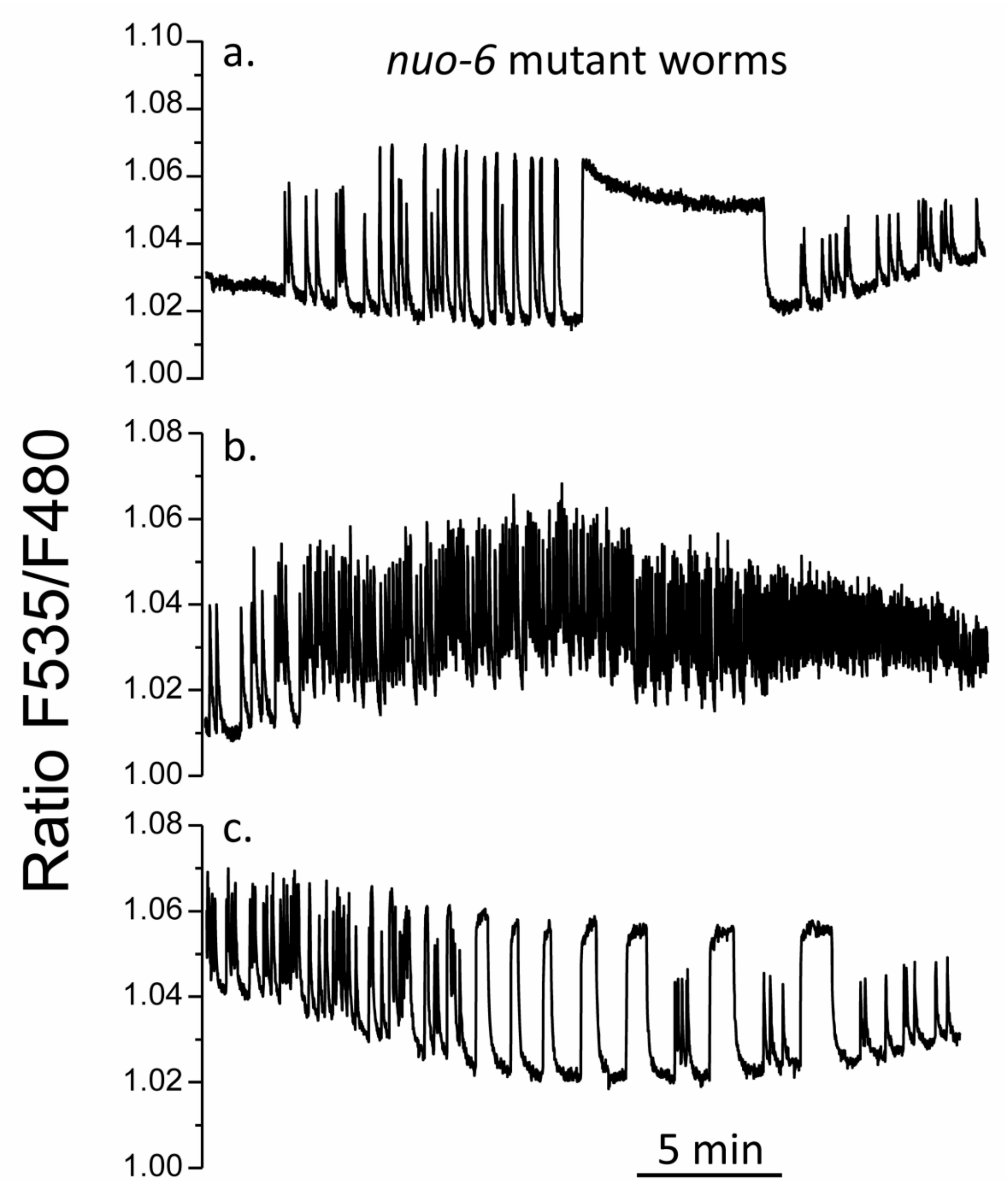

Figure S3. Representative examples of pharynx $[Ca^{2+}]_c$ recordings in 2-day old *nuo-6* mutant worms.
